# A Systematic Review and Meta-Analysis of the Efficacy of Buddy Taping Versus Reduction and Casting for Non-operative Management of Closed Fifth Metacarpal Neck Fractures

**DOI:** 10.7759/cureus.28437

**Published:** 2022-08-26

**Authors:** Mohamed B Mohamed, Christian N Paulsingh, Tarig H Ahmed, Zahir Mohammed, Trisha Singh, Mohamed S Elhaj, Nusyba Mohamed, Safeera Khan

**Affiliations:** 1 Internal Medicine, California Institute of Behavioral Neurosciences & Psychology, Fairfield, USA

**Keywords:** non-operative management, fracture, fifth metacarpal, boxer's fracture, plaster cast immobilization, buddy taping

## Abstract

A closed fifth metacarpal neck fracture is a frequently encountered upper limb fracture that occurs when the bone breaks right below the little finger's knuckle. At the moment, there is no agreement on the best way to treat these fractures. This research seeks to look at the efficacy of buddy taping versus reduction and casting for non-operative management of uncomplicated closed fifth metacarpal neck fractures. A systematic review of PubMed, Medical Literature Analysis and Retrieval System Online (MEDLINE), PubMed Central (PMC), and the Cochrane Library databases was carried out using the Preferred Reporting Items for Systematic Reviews and Meta-Analyses (PRISMA) guidelines to find relevant studies about buddy taping versus reduction and casting for non-operative management. Disabilities of the arm, shoulder, and hand (DASH) score; satisfaction score; visual analog scale (VAS); range of motion (ROM); strength; and other outcomes were reported in this study. We used Review Manager 5.4 (The Cochrane Collaboration, London, UK) for the meta-analysis. Seven trials with a total of 454 patients were considered in the review and four in the quantitative analysis. All the included studies were randomized controlled trials (RCTs).

Our study concluded that buddy taping was effective for improving pain, range of motion, and strength. The DASH score and satisfaction score didn't show any significant difference. Thus, we recommend the use of buddy taping rather than plaster and immobilization for the management of uncomplicated closed fifth metacarpal neck fractures.

## Introduction and background

Different fractures happen to the metacarpal bone, and one of these fractures is the fifth metacarpal neck fracture, also referred to as a boxer's fracture, which is a crack in the neck of the fifth metacarpal bone due to high velocity [1]. Metacarpal bone fractures constitute about 40% of hand fractures [2], and fifth metacarpal neck fractures account for 20% of hand fractures [3].

Fifth metacarpal neck fractures occur as a result of a direct collision or trauma to the clenched fist, resulting in apical dorsal angulation caused in part by stresses produced by interosseous muscle tension [4]. To diagnose a boxer's fracture and evaluate the degree of angulation, plain radiographs are the gold standard. It is necessary to obtain anteroposterior, lateral, and oblique views [4]. According to recent research, an initial diagnosis of a boxer's fracture may also be made using bedside ultrasound [5]. It's uncommon to utilize computed tomography (CT) scans to diagnose metacarpal fractures, but they can be useful in cases when there's a high degree of clinical suspicion of fracture but no plain radiographic evidence of a fracture [6].

Full immobilization with a plaster cast, functional taping with an elastic bandage, and a full dynamic treatment, in which the patient is advised to continue using the affected hand normally, have all been described as treatments for uncomplicated closed fifth metacarpal neck fractures where the fracture is an isolated injury with less than 70° of angulation, limited displacement, and no rotational deformity [3,7].

However, no previous study assessed the effectiveness of these interventions in reducing the pain and other outcomes related to the fracture. Therefore, we conducted this study to assess efficacy and outcomes related to closed fifth metacarpal neck fracture after being managed with either buddy taping or reduction and casting.

## Review

Methods

We conducted this systematic review according to the Cochrane Handbook for Systematic Reviews of Interventions [8]. Also, we performed this review by utilizing the Preferred Reporting Items for Systematic Reviews and Meta-analyses (PRISMA) statement [9]. This systematic review tests the hypothesis that patients with uncomplicated closed fifth metacarpal neck fractures being treated with buddy taping will have a favorable outcome compared to being treated with reduction and casting.

Search Strategy

We searched PubMed, Medical Literature Analysis and Retrieval System Online (MEDLINE), PubMed Central (PMC), and the Cochrane Library databases by using the keywords "Metacarpal Bones/injuries" (Majr) OR "Metacarpal Bones/surgery" (Majr) OR "Metacarpal Bones/therapy" (Majr) OR fifth metacarpal AND "Fractures, Bone/therapy" (Majr) AND "Casts, Surgical/therapy" (Majr) for all studies up to August 2021.

Eligibility Criteria and Study Selection

In this review, we selected only randomized controlled trials (RCTs), which compared non-operative treatment via buddy taping with reduction and casting in patients with a closed fifth metacarpal neck fracture. Any systemic review, meta-analysis, biomechanical study, technique articles, case series without a control group, case report, non-English studies, and animal studies were excluded. After getting articles from databases, we looked at the titles and abstracts and then read the full texts to find the final articles that were included.

Data Extraction

The following data was gathered from the included studies by the authors: (1) baseline characteristics of the studies' participants with fifth metacarpal neck fracture and summary of all included studies and (2) study outcomes at 12 weeks: disabilities of the arm, shoulder, and hand (DASH) score; satisfaction score; pain control measured by visual analog scale (VAS); range of motion (ROM); strength; and other outcomes in each included study.

Quality Assessment

We assessed the quality of included trials using the Cochrane risk of bias tool provided in the Cochrane Handbook for Systematic Reviews of Interventions (version 5.1.0) [8]. The domains included were (1) random sequence generation (selection bias), (2) allocation concealment (selection bias), (3) blinding of participants and personnel (performance bias), (4) outcome assessment (detection bias), (5) incomplete outcome data (attrition bias), and (6) other potential sources of bias. The reviewers judged the domains as "low risk," "high risk," or "unclear." The quality assessment table used was provided in part 2, chapter 2.5, of the same book [8].

Data Analysis

In the analysis, we used the mean difference (MD) to show the continuous data in a random-effect meta-analysis model using the inverse-variance method for continuous data. Missing standard deviation (SD) was calculated from median, standard error, or 95% confidence interval (CI) according to Altman [10]. In this analysis, we used Review Manager 5.4 (The Cochrane Collaboration, London, UK) for Windows.

Assessment of Heterogeneity

The heterogeneity of the pooled data was assessed by the I² and chi² tests presented in the forest plots. The chi² test measures the presence of significant heterogeneity, and the I² test quantifies the size of the heterogeneity in the pooled data. The interpretation of the results followed the recommendations of the Cochrane Handbook for Systematic Reviews of Interventions. The chi² test was considered significant with a P value of less than 0.1, while the I² test is interpreted as follows: 0-40%: not significant, 30-60%: moderate heterogeneity, and 50-90%: significant heterogeneity.

Results

Literature Search

**Figure 1:**
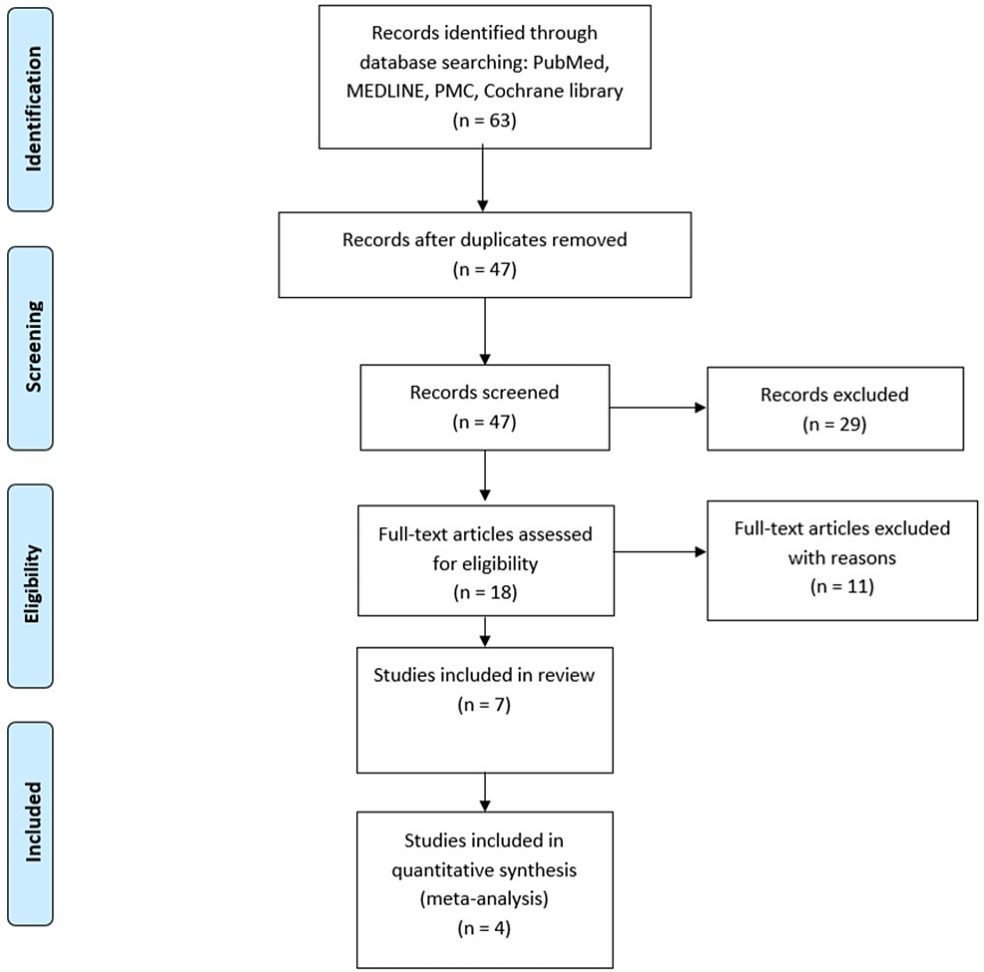
PRISMA 2020 flow diagram for systematic review PRISMA: Preferred Reporting Items for Systematic Reviews and Meta-Analyses; MEDLINE: Medical Literature Analysis and Retrieval System Online; PMC: PubMed Central

The literature search retrieved 47 citations after applying a filter for RCTs and removing duplications. After title and abstract screening, 18 articles were selected. We evaluated the full text of the selected studies. In the end, we could include seven studies in our review and four in our quantitative analysis (see Figure 1 for a PRISMA flow diagram).

Characteristics of the Included Studies and Quality Assessment

Our considered studies included a total of 454 patients that needed management for a fifth metacarpal neck fracture. The mean age ranged from 26.1 to 42.5 years across the studies. The baseline characteristics of the studies' participants are shown in Table 1.

**Table 1:** Baseline characteristics of participants with fifth metacarpal neck fracture

Study ID	Country	Sample size	Age, mean (years)	Males, %	Disabilities of the arm, shoulder, and hand (DASH) score	Pain visual analog scale (VAS) baseline, mm	Number in cast/splint group	Number in buddy/wrap/tape group	The average duration of treatment, week	Average follow-up, weeks	Maximum angulation without reduction
Braakman et al., 1998 [11]	The Netherlands	50	26.1	-	-	-	25	25	4	24	50°
Kuokkanen et al., 1999 [12]	Finland	29	29	26 (89.6%)	-	-	15	14	4	12	-
Statius Muller et al., 2003 [7]	The Netherlands	35	29	-	-	-	15	20	Splint, 3; wrap, 1	12	70°
Bansal et al., 2007 [13]	The United Kingdom	78	28	67 (85.9%)	-	-	40	38	3	12	70°
van Aaken et al., 2016 [14]	Switzerland and the United States	64	28.4	62 (96.9%)	(Quick DASH) Cast = 49.7 ± 21.8; buddy = 45.7 ± 18.0	Cast = 35.2 ± 22.7; buddy = 31.9 ± 19.9	27	37	3	16	70°
Pellatt et al., 2019 [15]	Australia	126	26.5	107 (85%)	(Quick DASH) Cast = 9.8 ± 16.8; buddy = 3.8 ± 8.4	-	64	62	3	12	70°
Martínez-Catalán et al., 2020 [3]	Spain	72	42.5	57 (79.1%)	-	Cast = 39.4 ± 16.5; buddy = 45.5 ± 18.9	38	34	3	9	-

A summary table of the quality assessment for the included randomized trials is shown in Table 2.

**Table 2:** Outcomes of fifth metacarpal neck fracture management ROM: range of motion; DASH: disabilities of the arm, shoulder, and hand; VAS: visual analog scale; EQ-5D-3L: EuroQol 5 Dimension 3 Level

Study ID	ROM		Strength		Other outcomes	
	Measurement	Favored	Measurement	Favored	Measurement	Favored
Braakman et al., 1998 [11]	Flexion deficit	Wrap	Pull	Wrap	-	-
	Extension deficient	Wrap	Torque	Wrap	-	-
			Pronation	Wrap	-	-
			Supination	Wrap	-	-
Kuokkanen et al., 1999 [12]	Metacarpophalangeal (MCP) joint	Wrap at four weeks	Grip	Wrap	Radiographic angulation (°)	Wrap
	Proximal interphalangeal joint	Wrap at four weeks			Bony union	Equivalent
Statius Muller et al., 2003 [7]	Metacarpophalangeal joint	Equivalent	-	-	Pain	Equivalent
	-	-	-	-	Satisfaction	Equivalent
	-	-	-	-	Mean radiographic angulation	Equivalent
Bansal et al., 2007 [13]	-	-	-	-	Time to return to work	Wrap
	-	-	-	-	Satisfaction scores (support given)	Wrap
	-	-	-	-	DASH score	Wrap
van Aaken et al., 2016 [14]	Flexion of the fifth MCP joint	Equivalent			Satisfaction	Equivalent
	Hyperextension of the fifth MCP joint	Equivalent	Grip	Equivalent	Quick DASH	Equivalent
	-	-	-	-	VAS	Equivalent
	-	-	-	-	Work leave days	Wrap
	-	-	-	-	Fracture angulation (°)	Equivalent
Pellatt et al., 2019 [15]	-	-	-	-	EQ-5D-3L score	Equivalent
	-	-	-	-	Pain score at one and 12 weeks	Equivalent
	-	-	-	-	Satisfaction score at one and 12 weeks	Equivalent
	-	-	-	-	Missed work, days	Equivalent
	-	-	-	-	Missed hobbies and sports, days	Equivalent
	-	-	-	-	Length of stay, minutes	Wrap
	-	-	-	-	Fracture angle	Equivalent
Martínez-Catalán et al., 2020 [3]	Fifth MCP abduction	Wrap	Grip	Equivalent	Work leave days	Wrap
	Flexion of the fifth MCP joint	Wrap			VAS	Wrap
	Extension of the fifth MCP joint	Wrap			DASH	Wrap
					Radiographic volar angulation	Equivalent

All the included RCTs showed moderate to high quality, and their risk of bias is shown in Table 3, seven RCT studies [3,7,11-15].

**Table 3:** Risk of bias for included studies

	Random sequence generation	Allocation concealment	Blinding of participants and personnel	Blinding of outcome assessment	Incomplete outcome data	Selective reporting	Other bias	Overall risk of bias
Martínez-Catalán et al., 2020 [3]	Low risk	High risk	High risk	High risk	Low risk	Low risk	High risk	High risk
Pellatt et al., 2019 [15]	Low risk	Low risk	High risk	High risk	Low risk	Low risk	Low risk	Low risk
van Aaken et al., 2016 [14]	Low risk	Unclear risk	High risk	High risk	Low risk	Low risk	Low risk	Low risk
Bansal et al., 2007 [13]	Unclear risk	Unclear risk	Unclear risk	Unclear risk	Unclear risk	Low risk	Low risk	Low risk
Statius Muller et al., 2003 [7]	Low risk	Unclear risk	High risk	High risk	Low risk	Low risk	Unclear risk	Low risk
Kuokkanen et al., 1999 [12]	Low risk	Unclear risk	High risk	High risk	Low risk	High risk	Unclear risk	High risk
Braakman et al., 1998 [11]	Low risk	Unclear risk	High risk	High risk	Low risk	High risk	Unclear risk	High risk

Findings From the Study

Study by Braakman et al. (1998) [11] found favorable effect of buddy taping than plaster immobilization in improving range of motion in flexion and extension deficient and in strength after assessing it using several measurements. Kuokkanen et al. (1999) [12] showed an improvement after using wrap buddy taping compared to casting in range of motion for the metacarpophalangeal joint and proximal interphalangeal joint at four weeks, strength, and radiographic angulation. Statius Muller et al. (2003) [7] found equivalent effect of both interventions for range of motion for the metacarpophalangeal joint, pain, satisfaction score, and mean radiographic angulation. Bansal et al. (2007) [13] showed a favorable effect for buddy taping over cast in time to return to work, satisfaction scores (support given), and DASH score. van Aaken et al. (2016) [14] showed an equivalent effect for both groups in most outcomes except for the number of work leave days, which showed fewer work leave days in the buddy taping group than in the cast group. Pellatt et al. (2019) [15] also showed an equivalent effect for both interventions except for length of stay, which showed less time in the buddy taping group. Martínez-Catalán et al. (2020) [3] found an improvement in range of motion, work leave days, visual analog scale (VAS), and DASH score for the buddy taping group compared to the cast group (Table 2). In this systematic review, different complications were noticed in five studies [3,7,11,12,15]. Further information is shown in Table 4.

**Table 4:** Complications of the fifth metacarpal neck fracture No.: number; MCP: metacarpophalangeal; PIP: proximal interphalangeal

Study ID	Complications	No./total no.	
		Splint/cast	Wrap/tape
Braakman et al., 1998 [11]	Residual symptoms at 24 weeks	9/25	8/23
Kuokkanen et al., 1999 [12]	-	-	-
Statius Muller et al., 2003 [7]	Return to work at six weeks	14/15	19/20
	Return to work at 12 weeks	15/15	20/20
Pellatt et al., 2019 [15]	Infection, nonunion, and delayed union	0/64	0/62
Martínez-Catalán et al., 2020 [3]	Complications at three weeks	10/38	3/34
	Complications at nine weeks	9/38	2/34
	MCP and PIP joint stiffness	9/38	2/34

DASH Score at 12 Weeks

**Figure 2:**
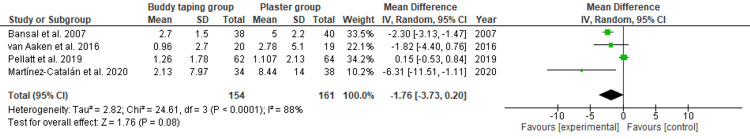
DASH score in 12 weeks DASH: disabilities of the arm, shoulder, and hand; SD: standard deviation; CI: confidence interval; df: degrees of freedom Bansal et al., 2007 [13]; van Aaken et al., 2016 [14]; Pellatt et al., 2019 [15]; Martínez-Catalán et al., 2020 [3]

The pooled effect estimate [3,13-15] showed no statistically significant difference between buddy and cast in DASH score at 12 weeks (MD = -1.76, 95% CI {-3.73, 0.20}, P = 0.08) (Figure 2).

**Figure 3:**
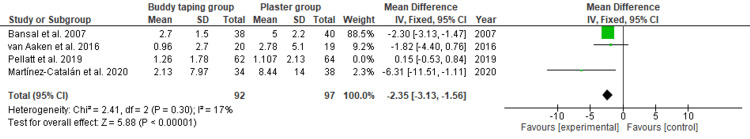
DASH score in 12 weeks after removing the study of Pellatt et al. (2019) DASH: disabilities of the arm, shoulder, and hand; SD: standard deviation; CI: confidence interval; df: degrees of freedom Bansal et al., 2007 [13]; van Aaken et al., 2016 [14]; Pellatt et al., 2019 [15]; Martínez-Catalán et al., 2020 [3]

The pooled studies were heterogeneous (P < 0.0001, I² = 88%), and the detected heterogeneity was solved after excluding Pellatt et al. (P = 0.30, I² = 17%); then, the results became significant, favoring buddy taping over cast (MD = -2.35, 95% CI {-3.13, -1.56}, P < 0.00001) (Figure 3).

Satisfaction Score at 12 Weeks

**Figure 4:**
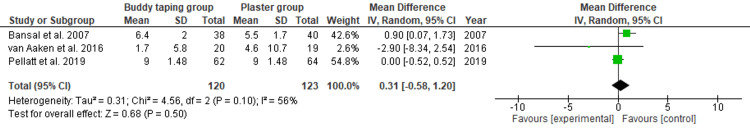
Satisfaction score after 12 weeks SD: standard deviation; CI: confidence interval; df: degrees of freedom Bansal et al., 2007 [13]; van Aaken et al., 2016 [14]; Pellatt et al., 2019 [15]

The pooled effect estimate [13-15] showed no statistically significant difference between the two interventions for satisfaction score at 12 weeks (MD = 0.31, 95% CI {-0.58, 1.20}, P = 0.50) (Figure 4). Pooled results were heterogeneous (P = 10, I² = 56%), and the detected heterogeneity could not be solved.

VAS at 12 Weeks

**Figure 5:**
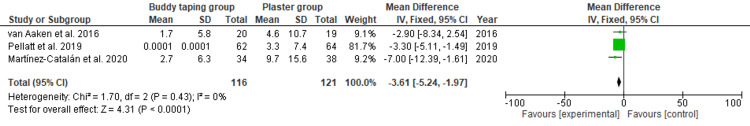
VAS pain score at 12 weeks VAS: visual analog scale; SD: standard deviation; CI: confidence interval; df: degrees of freedom van Aaken et al., 2016 [14]; Pellatt et al., 2019 [15]; Martínez-Catalán et al., 2020 [3]

The pooled effect estimate [3,14,15] showed a statistically significant difference in pain VAS between buddy and plaster immobilization favoring buddy taping over casting (MD = -3.61, 95% CI {-5.24, -1.97}, P < 0.0001) (Figure 5). Pooled studies were homogenous (P = 0.43, I² = 0%).

Discussion

This meta-analysis compared buddy taping with plaster immobilization or casting for managing patients with an uncomplicated closed fifth metacarpal neck fracture. We found that buddy taping is effective in reducing pain after 12 weeks of management. Also, several studies showed a better outcome for buddy taping than casting in range of motion and strength. Other outcomes across all included studies showed either an equal effect or an advantage for using buddy taping over casting. Regarding complications and the number of days to return to work, buddy taping was better than casting in the previously mentioned variables.

Range of motion was measured in different ways in the included studies and showed effectiveness for buddy taping over casting in three studies [3,11,12] and equal effect in two studies [7,14]. However, the strength of the joint was found to be better in the buddy taping/wrap group in two studies [11,12], and there was an equal effect for grip in two studies [3,14]. These outcomes are presented in a table only without the ability to conduct a meta-analysis.

DASH is an assessment tool consisting of 30 questions to assess symptoms and physical functioning in the upper limbs of participants [16] and is one of the most reliable patient-reported outcome measures [17]. Due to long questions being asked to participants, another shortened tool from DASH was developed, named Quick DASH [18]. The meta-analysis for the DASH score showed no difference between the two interventions applied to each group [3,13-15], and it became significant after removing one study to solve heterogeneity [15], and this showed low disability in the buddy taping group, which helped in fast recovery after fractured fifth metacarpal. Satisfaction about the intervention was measured in some studies using a score from zero to 10 [13-15], and we didn't find any difference in satisfaction after 12 weeks of the intervention. This means that satisfaction was not different between the two interventions.

Pain is considered one of the important factors taken into consideration after the fifth metacarpal neck fracture. Our meta-analysis found that buddy taping is better than reduction and casting (MD = -3.61, 95% CI {-5.24, -1.97}, P < 0.0001) in reducing pain after 12 weeks [3,14,15], and this will lead to fast recovery from the fracture and a return to normal activity. Complications were considered one of the concerns surgeons face after fractures. Our study showed a low number of patients with complications or joint stiffness when using buddy taping [3].

Our meta-analysis is considered the first one to compare and assess the effectiveness of buddy taping versus reduction and casting for non-operative management of the fifth metacarpal neck fracture. It provides us with valuable information that would help hand surgeons choose buddy taping in managing fifth metacarpal neck fractures due to its favorable outcome and less pain compared with casting. Furthermore, this meta-analysis only included RCTs, which strengthens our findings and could be used in guidelines for managing uncomplicated closed fifth metacarpal neck fracture.

Aside from these advantages, we encountered several limitations. The involvement of a small number of participants across all included RCTs is one of these limitations. In addition, all the included studies had different follow-up periods. We also couldn't do a meta-analysis for some outcomes because numerous outcomes were not observed in some trials.

## Conclusions

Buddy taping was found to be more successful than reduction and casting for addressing pain, range of motion, and strength. In accordance with the findings of our study, we recommend buddy taping for the management of uncomplicated closed fifth metacarpal neck fracture. Additional research should include a larger sample size and a longer duration of follow-up.
